# The Role of Pre-existing Cross-Reactive Central Memory CD4 T-Cells in Vaccination With Previously Unseen Influenza Strains

**DOI:** 10.3389/fimmu.2019.00593

**Published:** 2019-04-04

**Authors:** Mikalai Nienen, Ulrik Stervbo, Felix Mölder, Sviatlana Kaliszczyk, Leon Kuchenbecker, Ludmila Gayova, Brunhilde Schweiger, Karsten Jürchott, Jochen Hecht, Avidan U. Neumann, Sven Rahmann, Timm Westhoff, Petra Reinke, Andreas Thiel, Nina Babel

**Affiliations:** ^1^Institute for Medical Immunology, Charité University Medicine Berlin, Berlin, Germany; ^2^Berlin-Brandenburg Center for Regenerative Therapies, Charité University Medicine Berlin, Berlin, Germany; ^3^Labor Berlin-Charité Vivantes GmbH, Berlin, Germany; ^4^Center for Translational Medicine, Immunology and Transplantation, Marien Hospital Herne, Ruhr University Bochum, Herne, Germany; ^5^Genome Informatics, Institute of Human Genetics, University Hospital Essen, University of Duisburg-Essen, Essen, Germany; ^6^Applied Bioinformatics, Tübingen University, Tübingen, Germany; ^7^Bogomolets National Medical University, Kyiv, Ukraine; ^8^Robert-Koch Institute, Berlin, Germany; ^9^Centre for Genomic Regulation (CRG), The Barcelona Institute of Science and Technology, Barcelona, Spain; ^10^Universitat Pompeu Fabra (UPF), Barcelona, Spain; ^11^Institute of Environmental Medicine, German Research Center for Environmental Health, Helmholtz Zentrum München, Augsburg, Germany; ^12^Department of Internal Medicine, Marien Hospital Herne, Ruhr University Bochum, Herne, Germany; ^13^Department of Nephrology and Intensive Care, Charité University Medicine Berlin, Berlin, Germany

**Keywords:** influenza vaccination, vaccination efficacy, pre-existing cross-reactive T-cells, central memory T-cell, clonotype diversity

## Abstract

Influenza vaccination is a common approach to prevent seasonal and pandemic influenza. Pre-existing antibodies against close viral strains might impair antibody formation against previously unseen strains–a process called original antigenic sin. The role of this pre-existing cellular immunity in this process is, despite some hints from animal models, not clear. Here, we analyzed cellular and humoral immunity in healthy individuals before and after vaccination with seasonal influenza vaccine. Based on influenza-specific hemagglutination inhibiting (HI) titers, vaccinees were grouped into HI-negative and -positive cohorts followed by in-depth cytometric and TCR repertoire analysis. Both serological groups revealed cross-reactive T-cell memory to the vaccine strains at baseline that gave rise to the majority of vaccine-specific T-cells post vaccination. On the contrary, very limited number of vaccine-specific T-cell clones was recruited from the naive pool. Furthermore, baseline quantity of vaccine-specific central memory helper T-cells and clonotype richness of this population directly correlated with the vaccination efficacy. Our findings suggest that the deliberate recruitment of pre-existing cross-reactive cellular memory might help to improve vaccination outcome.

## Introduction

Influenza infection is a major cause of acute respiratory infections (1, 2). While healthy individuals manage the infection efficiently, several groups, including elder, immunosuppressed, and chronically ill individuals, have a significant risk of prolonged and complicated infection course and high mortality (2, 3).

Vaccination with trivalent inactivated vaccine (TIV) or live attenuated vaccine (LAV) is the common approach to raise protective antibody titers against influenza and is generally accepted as the most relevant protection factor (4, 5). However, it is not rare that the post vaccination antibody levels are insufficient. Even though several clinical conditions are associated with low vaccination efficacy (e.g., chronic inflammatory and metabolic disorders, immune deficiencies), the scenario of insufficient or failed vaccination also affects the healthy population (6–8).

The exact prerequisites and correlates of efficient vaccination are not completely understood so far but have been attributed to the vaccine origin, its composition and application mode (9–14). In case of a primary immune response the contact with previously unseen pathogenic antigens leads to an inflammatory process and the recruitment of T- and B-cells from naive pools. Besides generation of effector cells this leads to formation of the immune memory, both cellular and humoral. In case of a secondary immune response, pre-existing memory B- and T-cells promptly proliferate, differentiate and perform numerous effector functions, resulting in a rapid raise of antibodies titers and pathogen clearance. For influenza however, the situation is somewhat special. The previous contacts with influenza leave long-lasting and sometimes life-long cellular and humoral immunity. However, due to antigenic drift and shift new viral strains are continuously created which are no longer recognized by the pre-existing memory, what helps the virus to bypass the pre-existing immunity (15–18). The exact role of pre-existing immune memory in the development of sufficient protection against novel epitopes is not clear, yet. Numerous findings indicate that it can be detrimental and lead to impaired formation of neutralizing antibody against previously unseen influenza strains. This phenomenon known as the original antigenic sin (OAS) was initially linked to the pre-existing cross-reactive antibodies and cognate memory B-cells (19, 20). However, the role of pre-existing cross-reactive T-cells in an insufficient and failed immune response against novel influenza strains was inferred from the studies on Dengue virus and mouse LCMV (21, 22). This was further strengthened by several reports on the suppression of naive and follicular influenza-specific helper T-cells by the pre-existing cross-reactive memory (23, 24). However, new findings show that pre-existing influenza-specific memory, both cellular and humoral, is not always detrimental but on the contrary might be helpful in terms of vaccination efficacy and protection against natural infection (25, 26). One report showed that the pre-existing cross-reactive memory CD4 specific to highly conserved internal influenza virus proteins are sufficient to alleviate influenza infection in a human inoculation model (27). However, data on the role of pre-existing memory against highly variable hemagglutinin (HA) and neuraminidase (NA) induced by vaccination are very limited.

The goal of the current study was to elucidate the generation of influenza-specific helper T-cells upon vaccination with novel, previously unexperienced strains and to unravel their role in the formation of humoral immunity against novel influenza strains.

## Materials and Methods

### Study Cohort

A total of 15 healthy adult individuals between 24 and 64 years old were involved in the study. The including criteria were as follows: 18 years or older, no previous influenza vaccination with the strains from the current composition (seasonal influenza vaccine 2011/2012) and/or no confirmed influenza infections in the past three years, no acute or chronic diseases, no known allergy to vaccine components, no pregnancy, good general health condition, written informed consent.

### Vaccination and Sample Collection

The vaccination was performed intramuscularly by a study physician with the trivalent influenza vaccine (Mutagrip 2011/2012 Sanofi-Pasteur). The vaccine was composed of A/California/7/2009 (H1N1), A/Perth/16/2009 (H3N2), B/Brisbane/60/2008 according to WHO recommendation. 50 ml venous blood was drawn at day 0, 7, 14, and 21 post vaccination using Lithium-Heparin Vacutainers (BD Biosciences) and processed immediately.

### Hemagglutination Inhibition Assay

Influenza-specific antibody titers were measured by a standard hemagglutination inhibition (HAI) assay, using vaccine strains (s. vaccine composition) and turkey hen erythrocytes (28). Baseline (day 0) and post vaccination (day 21) sera were tested simultaneously in duplicates and the antibody titers estimated. Baseline seronegativity was defined by a HAI titer <10 (29). For statistical evaluation the combined vaccination efficacy for three vaccine components was calculated as the sum of the binary logarithm fold change (ΔLF) between day 21 and baseline according to the formula:

$$\begin{array}{l} \Delta LF=\overset{3}{\underset{c=1}{\sum }}\log_{2}\left(\frac{titer\left(c,day21\right)}{titer\left(c,day0\right)}\right), \end{array}$$

where the sum ranges over the three components *c*.

### PBMC Preparation

Peripheral blood mononuclear cells (PBMCs) were isolated by gradient centrifugation with Ficoll-Paque Plus (GE Healthcare). PBMCs were re-suspended in complete medium (RPMI/10%FCS/Penicillin/Streptomycin, all from Gibco).

### Flow Cytometric Assessment and Isolation of Influenza-Specific Helper T-Cells

Frequency, cytokine production and phenotype analysis of the influenza-specific helper T-cells was done after overnight PBMC stimulation with the vaccine (at least 10 μg/mL of HA from every strain). As negative and positive controls, PBMC were incubated alone or with staphylococcal enterotoxin B (1 μg/ml, Sigma-Aldrich). Brefeldin A was added after 2 h of stimulation (10 μg/mL, Sigma-Aldrich). At the end of the stimulation PBMC were harvested, stained for surface, and intracellular markers using FACS-Lysing and FACS-Perm Solution (BD Biosciences), and analyzed on BD Fortessa flow cytometer (BD Biosciences).

Influenza-specific helper T-cells were isolated after overnight PBMC stimulation with the vaccine (10 μg/mL of HA from every strain) and human anti-CD40 antibodies (clone HB14, Miltenyi Biotec). Live sorting was done on BD FACS Aria (BD Biosciences) with sorting strategy provided in Figure S2. The following subsets were enriched with high purity: naive (CD45RA+CCR7+), central memory (CM, CD45RA-CCR7+) and effector (Eff, CD45RA-CCR7-). Following antibodies were used for the cytometric analysis and sorting: CD3 eFluor 650 (HIT3a, eBioscience), CD4 QDot 565 (OKT4, Biolegend, in-house fluorochrome coupling), CD8 QDot 525 (RPT-T8, Biolegend, in-house fluorochrome coupling), CCR7 FITC (G043H7, Biolegend), CD45RA eFlour 605 (HI100, eBioscience), CD154 APC/Cy7 (24-31, Biolegend), CD69 Pe/Cy5 (FN50, Biolegend), TNFa Pacific Blue (Mab11, Biolegend), IFNg Alexa Fluor 700 (B27, Biolegend), IL2 Pe (MQ1-17H12, Biolegend), IL4 Pe/Cy7 (MP4-25D2, Biolegend), IL17 PerCP/Cy5.5 (BL168, Biolegend), CD19 V500 (HIB19, BD Biosciences), CD27 PerCP-Cy5.5 (M-T271, Biolegend), IgD FITC (IA6-2, BD Biosciences), CD20 eFluor 650 (2H7, eBioscience), CD38 Alexa Fluor 700 (HB-7, Biolegend), CD2/3/4/14/15/34/56/61/235a-biotin (as part of Pan B Cell Isolation Kit, Miltenyi Biotec), anti-biotin-Vio Blue (Bio3-18E7, Miltenyi Biotec). Peripheral blood plasmablasts were gated as CD27++CD38++CD20low/- cells among Lineage-CD19low/+ population (Figure S1). Absolute cell counts in peripheral blood were estimated as previously described (30). Detailed information on the sorted influenza-specific CD4 T-cells is provided in Table S1.

### Clonotype Analysis

The clonotype analysis was performed based on NGS-sequencing of the TCRβ chain of the FACS-enriched influenza-specific subsets. The detailed method description with primer sequences and amplification parameters can be found in the original publication (31, 32). Briefly, DNA was isolated using AllPrep DNA Micro Kit (QIagen) and the recombined TCRβ locus was amplified and processed using Illumina NGS platform. The raw sequencing data were deposited at Sequence Read Archive (SRA) with the following BioProject ID: PRJNA445234.

The raw sequences were processed with subsequent clone grouping on the nucleotide level using our free open-source clonotyping platform IMSEQ with analysis parameters provided in the supplementary Method Information (33). Detailed information on recovered sequencing reads is provided in Table S1. For the clonotype richness and overlap analysis, samples with less than 1,000 raw sequencing reads were discarded. In order to increase sensitivity of the clonotype analysis, clonotypes from the memory subsets at baseline (CM and Eff day 0) were grouped as a common pre-existing memory. The unique clones from the naive and common memory at baseline were tracked into the memory subsets post vaccination and the cumulative frequencies of the corresponding clones were calculated. Clonotype richness was assessed as the number of unique clonotype after sample size normalization. For this reason, subsets were size-normalized to 40,000 raw sequencing reads (corresponding to the size of the smallest analyzed sample) and the unique clones grouped. The number of unique clones per normalized sample represented the value of clonotype richness.

### Flow Cytometry and Statistical Data Analysis

FACS data were analyzed with FlowJo 9.9.3 (TreeStar).

Statistical analysis was performed using GraphPad Prism with following hypotheses defined beforehand:

1. Serologically exposed and non-exposed cohorts show different kinetics of peripheral blood B- and influenza-specific CD4 T-cells,
2. Pre-existing influenza-specific T-cells define vaccination efficacy in the serologically non-exposed cohort,
3. Origin of influenza-specific CD4 T-cells post vaccination: baseline naive or cross-reactive memory,
4. Clonotype diversity/richness of the pre-existing influenza-specific CD4 T-cells define vaccination efficacy in the serologically non-exposed vaccinees.

Normality distribution was assessed by D'Agostino-Pearson omnibus or Shapiro-Wilk normality test. In case of normal distribution parametric *t* test and Pearson correlation were calculated; otherwise Mann-Whitney test and Spearman correlation were performed. Multiple comparisons were adjusted using the Holm-Sidak approach. *P*-values< 0.05 were considered significant and designated as following: <0.05 as ^*^, <0.01 as ^**^ and <0.001 as ^***^.

## Results

First, we assessed the vaccination efficacy in the cohort of 15 healthy individuals anamnestically not exposed to the natural influenza or the seasonal vaccination in the previous 3 years. This way subjects with no recent definite contact with influenza were preselected. However, despite preselection strategy, further serology analysis showed preformed hemagglutination inhibiting (HI)-antibodies to one or several viral strains in 7 out of 15 study participants at baseline. The vaccinees were therefore stratified into HI-positive and -negative groups according to the baseline antibody titers (Table 1). Of note, there were no non-responders in the study. All individuals developed protective antibody titers upon vaccination.

**Table 1:** Humoral responses to seasonal influenza vaccine assessed as titers of neutralizing antibodies.

**Donor ID**	**Age**	**Gender**	**California D0**	**Brisbane D0**	**Perth D0**	**California D21**	**Brisbane D21**	**Perth D21**	**ΔLF**
#30	26	M	1.00	1.00	1.00	8.32	4.91	4.32	14.55
#37	56	F	4.32	3.32	6.32	5.32	3.32	7.32	2.00
#38	30	M	1.00	1.00	1.00	1.00	6.32	3.32	7.64
#39	59	F	1.00	1.00	1.00	1.00	6.32	6.32	10.64
#40	61	M	1.00	1.00	1.00	4.32	7.91	4.32	13.55
#41	57	M	1.00	3.32	1.00	6.32	5.32	5.91	12.23
#42	64	F	1.00	1.00	1.00	1.00	5.32	11.32	14.64
#43	64	M	1.00	5.32	1.00	5.32	6.32	6.32	10.64
#45	26	M	7.32	5.32	1.00	8.32	9.32	5.32	9.32
#47	29	M	1.00	1.00	1.00	5.32	7.91	9.32	19.55
#51	26	M	1.00	1.00	1.00	7.91	4.32	6.91	16.14
#52	24	M	7.32	6.32	1.00	7.32	6.32	4.32	3.32
#53	26	F	1.00	1.00	1.00	9.91	7.32	7.32	21.55
#54	62	F	1.00	4.32	4.32	6.32	6.32	10.32	13.32
#55	29	F	5.32	4.32	4.32	7.32	7.32	7.91	8.58

*Neutralizing antibodies were assessed in HIA at baseline and day 21 post vaccination. Data are shown as binary logarithm of the corresponding dilution titers. ΔLF represents the summary serology change for three influenza strains included in the current vaccine composition*.

### HI-Negative Donors Develop a Higher Plasmablast Response Post Vaccine Application

**Figure 1:**
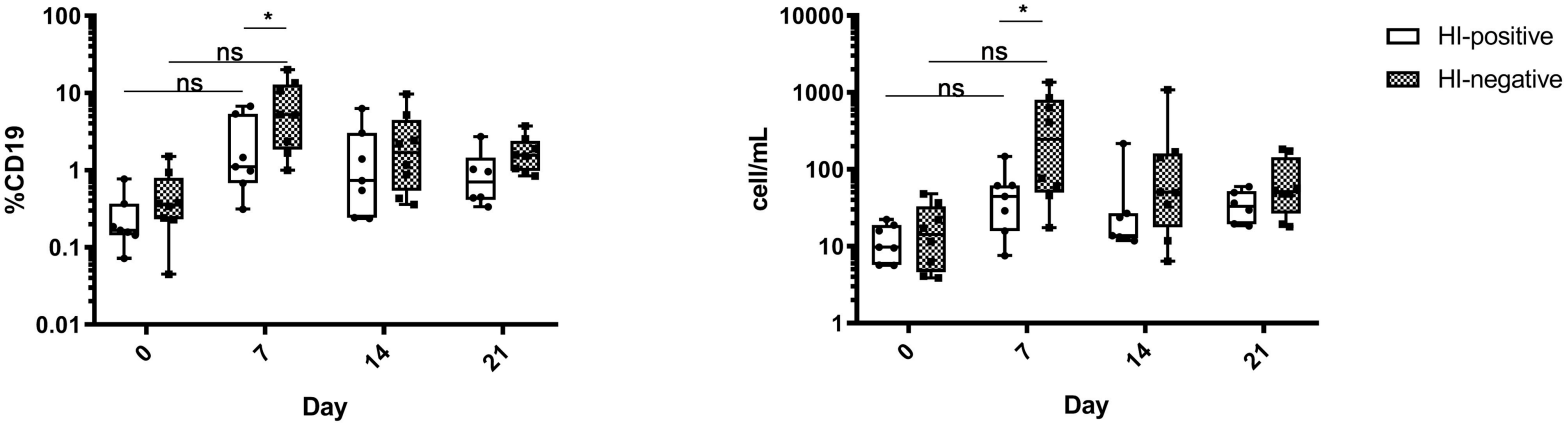
Enhanced peripheral blood plasmablast response in the serologically naive group after vaccine application. Peripheral blood plasmablasts (PB) were defined as CD27++CD38+ cells among CD19+/low population as relative frequencies and absolute cell numbers per mL peripheral blood. Analyses were performed at baseline and different time points post vaccination in both HI-negative (*n* = 8) and HI-positive (*n* = 7) groups. Parametric *t* tests with the Holm-Sidak approach for multiple comparisons were performed. The box plots show median with 25th to 75th percentiles and min to max range (whiskers). *P*-values are designated as following: <0.05 as ^*^, <0.01 as ^**^ and <0.001 as ^***^. The applied gating strategy is provided in Figure S1.

In order to determine any difference in the B-cell kinetics in two serological groups, we analyzed peripheral blood B-cells including plasmablasts (PB) at baseline and day 7, 14, and 21 post vaccination by flow cytometry. We did not observe any relevant changes in B-cell populations except PB. These were defined as CD27++CD38++CD20low/- among CD19+/low population (Figure S1) and analyzed as relative frequencies and absolute counts per mL whole blood. Significant PB rise at day 7 post vaccination was present in both groups (Figure 1; HI-positive group *p* < 0.01; HI-negative group *p* < 0.001 and *p* < 0.01 analyzed as frequencies and counts, correspondingly). The HI-positive group showed less pronounced changes at day 7, and the HI-negative group had significantly higher PB (*p* < 0.05 for both frequencies and absolute counts). Though the analyses were done on the whole blood level without further determination of B-cell antigen specificity, the observed population reflects kinetics of the influenza-specific PB, as previously shown (34, 35).

### Influenza-Specific Central Memory CD4 T-Cells Influence the Vaccination Outcome in HI-Negative Individuals

In order to analyze the role of CD4 T-cells, peripheral blood samples from both groups were stimulated with the vaccine and analyzed by means of multiparameter flow cytometry using markers of antigen-specific stimulation (31, 36). Analyses were performed at baseline and day 7, 14, and 21 post vaccination as CD4 T-cell frequencies and absolute counts (Figure S2).

**Figure 2:**
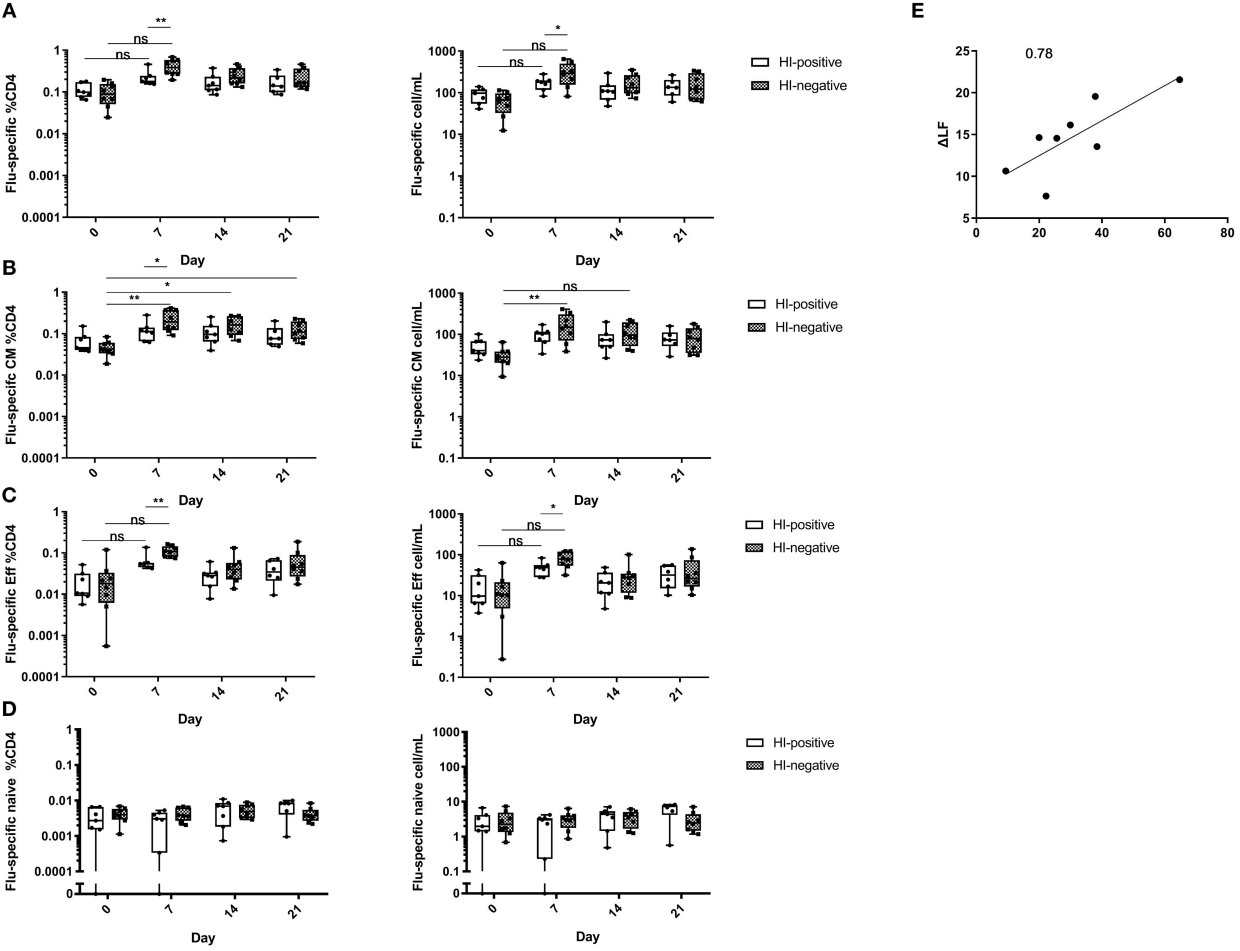
Influenza-specific CD4 T-cells with CM phenotype define the vaccination efficacy in the serologically naive cohort. **(A)** Vaccine-specific helper T-cells were analyzed in both serologically experienced (*n* = 7) and naive (*n* = 8) cohorts based on expression of CD154 and CD69, the cytokine-independent markers of antigen-specific CD4 T-helper activation. Influenza-specific helper T-cells were further analyzed based on CCR7 and CD45RA allowing discrimination of cell with CM **(B)**, Eff **(C)**, and naive phenotype **(D)**. CM helper T-cells were defined as CCR7+CD45RA-, Eff as CCR7-CD45RA- and naive as CCR7+CD45RA+. Relative frequencies among CD4 helper T-cells and absolute cell numbers per mL peripheral blood are shown. Parametric *t* tests with Holm-Sidak approach for multiple comparisons were performed. The box plots show median with 25th to 75th percentiles and min to max range (whiskers). *P*-values are designated as following: <0.05 as ^*^, <0.01 as ^**^ and <0.001 as ^***^. The applied gating strategy is provided in Figure S2. **(E)** Pearson correlation analysis of pre-existing vaccine-specific CD4 T-cells with CM phenotype in serologically unexperienced cohort at baseline (*n* = 8) analyzed as absolute cell numbers per mL peripheral blood and post-vaccination antibody titer increase. R, Pearson correlation coefficient. The line represents the best linear fit.

As anticipated, the HI-positive cohort showed pre-existing influenza-specific helper T-cells at baseline. This was also the case in the HI-negative individuals (Figure 2A). The kinetics analysis showed a significant increase of vaccine-specific CD4 T-cells in both groups with the peak at day 7 post vaccination (HI-positive group *p* < 0.05 for frequencies and absolute counts; HI-negative group *p* < 0.001 for frequencies and *p* < 0.01 for absolute counts) and a steady decline at later time points (Figure 2A). Of interest, the HI-negative subjects revealed a significantly higher magnitude of influenza-specific helper T-cells at the peak of vaccine-induced response as compared to HI-positive cohort. While no differences between serological groups were found at baseline and decline, at day 7 the HI-negative group showed a significantly higher vaccine-specific response (*p* < 0.01 for frequencies and *p* < 0.05 for cell counts).

We next analyzed the differentiation status of influenza-specific CD4 T-cells before and after immunization. Using CCR7 and CD45RA the differentiation status of T-cells can be assessed with division into following subsets: naive (CD45RA+CCR7+), central memory (CM, CD45RA-CCR7+), effector (Eff, CD45RA-CCR7-), and terminally differentiated memory T-cells (T_EMRA_, CD45RA+CCR7-). Our data showed that the majority of vaccine-specific T-cells at baseline were of memory phenotype (Figures 2B–D). In both serological groups, CM dominated over Eff. Surprisingly, both groups also revealed influenza-specific T-cells with naive phenotype at baseline (Figure 2D). Though in absolute minority as compared to memory subsets, naive cells were present in all participants.

The kinetics of vaccine-specific CM CD4 T-cells in the HI-positive group showed no significant changes. In the HI-negative group on the contrary, the changes were highly pronounced. Compared to baseline, influenza-specific CM CD4 T-cells showed a significant increase with the peak at day 7 with further decline (Figure 2B; *p* < 0.001 and *p* < 0.01 between baseline and day 7 and 14, respectively, for both frequencies and absolute counts; *p* < 0.05 between baseline and day 21 for frequency analysis).

Kinetics of vaccine-specific Eff CD4 T-cells resembled the pattern of unseparated influenza-specific T-cells with the peak at day 7 and a steady decline thereafter (Figure 2C; HI-positive group *p* < 0.01 and *p* < 0.05 for frequencies and counts; HI-negative group *p* < 0.01 for both analyses).

Unexpectedly, vaccine-specific T-cells with naive phenotype did not show any relevant changes in the course of immunization and were still present post vaccination (Figure 2D). These cells showed a truly naive nature as Boolean gating revealed low IL2 and no effector cytokine production. Memory influenza-specific T-cells on the contrary produced all measured effector cytokines (Figures S3A,B). Some donors revealed vaccine-specific T_EMRA_ CD4 T-cells, however, at extremely low frequencies. This cell subset was therefore not analyzed further (Figure S2).

We further wondered, which factors influenced the establishment of influenza-specific humoral immunity. Thus, we performed a correlation analysis between the amount of influenza-specific helper T-cells, either complete or further separated based on different differentiation status, and the degree of humoral response. We found that in the HI-negative group the absolute counts of influenza-specific CM T-cells at baseline correlated significantly with the change in vaccine-specific antibody titer (Figure 2E; Pearson *R* = 0.78, adjusted *p* = 0.02). We could not identify other correlations in the HI-negative group; there were no correlations in the HI-positive group. Taken together, the data presented here show that the number of CM T-cells correlates with the vaccination efficacy in H-negative vaccinees.

### Influenza-Specific Helper T-Cells Post Vaccination Are Predominantly Recruited From the Pre-existing Memory

Both serological groups showed efficient vaccination as reflected by sufficient titers increase post vaccination (Table 1). The influenza-specific T- and B-cells in the HI-positive group were responsible for the sufficient cellular and humoral immunity resulting in increased HI-titers. In the HI-negative group, on the contrary, the role of the pre-existing cross-reactive memory T-cells in the vaccination process was not clear and for this reason we aimed to investigate to which extent these pre-existing T-cells contributed to the formation of influenza-specific T-cell memory as opposed to naive vaccine-specific T-cells.

**Figure 3:**
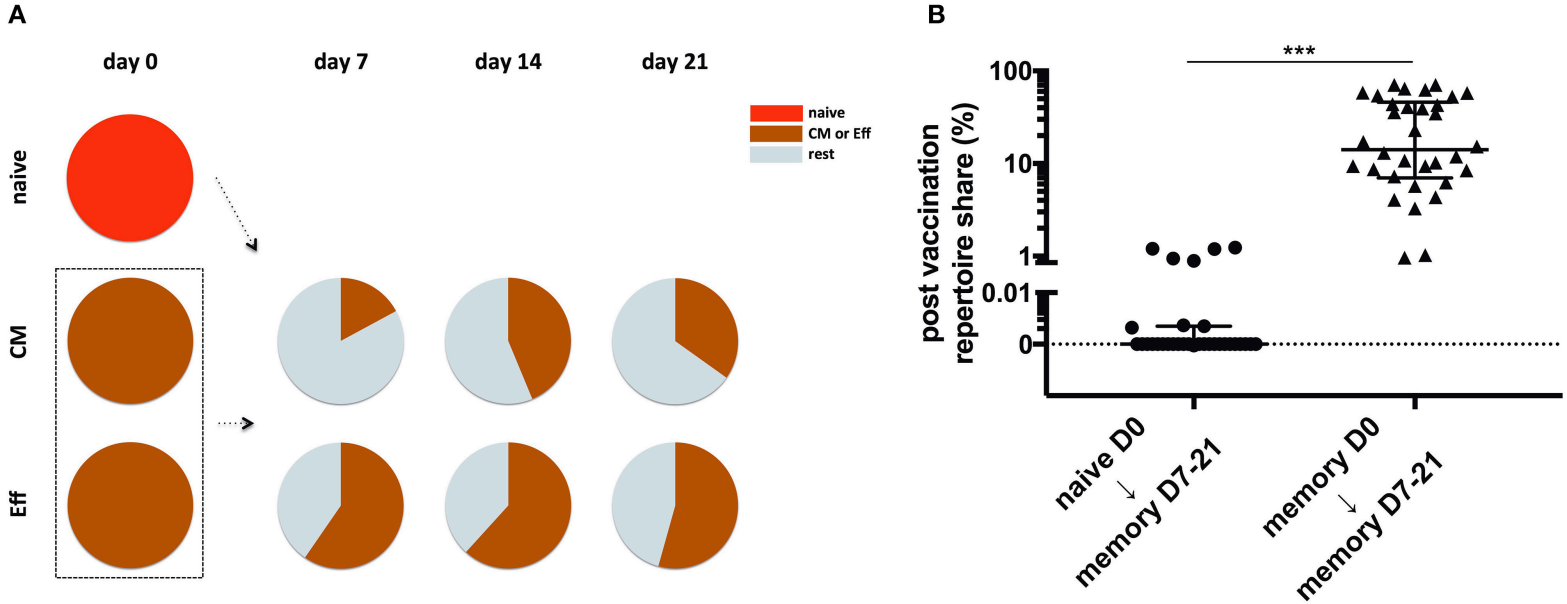
Post vaccination influenza-specific helper T-cell repertoires in the serologically unexposed group are formed predominantly from the pre-existing cross-reactive memory and not the naive T-cells. In the HI-negative cohort influenza-specific clonotypes from the baseline naive and memory subsets (CM and Eff) were tracked in the memory post vaccination and cumulative frequencies of the clonotypes with different origin (either naive or memory) were determined. **(A)** Schematic representation of the performed analysis. Single clonotypes from naive and common pre-existing memory were tracked in post vaccination subsets; cumulative frequencies of the corresponding clonotypes were estimated. **(B)** Cumulative frequencies of the influenza-specific clonotypes post-vaccination (*n* = 40) originating from either naive (*n* = 6) or pre-existing cross-reactive memory subsets (*n* = 14) at baseline. ^*^p < 0.05, ^**^p < 0.01, and ^***^p < 0.001. Detailed information on sorted cell populations and sequencing outcome is provided in Table S1.

For this purpose, subsets of vaccine-specific T-cells based on differentiation status were FACS-sorted at baseline and all analysis points post vaccination. The T-cell receptor (TCR) repertoires of all sorted populations were analyzed by sequencing of the TCRβ chain on the nucleotide level. As T-cells originating from the same progenitor bear identical TCR on the cell surface, it can be used as a cellular identifier to track and thus elucidate the origin of T-cells with different phenotypic status, inter-subset dynamics and/or tissue distribution as previously demonstrated (31, 32). In order to define the origin of the influenza-specific CD4 T-cells post vaccination, unique clonotypes from the sorted baseline naive or pre-existing cross-reactive memory subsets (CM and Eff) were tracked post vaccination at memory subsets and the cumulative repertoire shares for the found clonotypes were calculated (schematically shown in Figure 3A). The analysis revealed that the influenza-specific clonotypes were predominantly recruited from the pre-existing cross-reactive memory and that these clonotypes constituted absolute majority of the vaccine-induced helper T-cells. Tracking naive clonotypes from day 0 in post vaccination repertoires revealed shared clonotypes of about 1% in only six out of 34 comparison pairs. The remaining pairs showed either neglectable clonotype share or could not reveal any single naive clonotype in post vaccination memory (Figure 3B). The pre-existing cross-reactive memory, on the contrary, contributed significantly higher to the post vaccination repertoires constituting up to 80% of the memory clonotypes (*p* < 0.001; Figure 3B). Based on these observations, we conclude, that the influenza-specific helper T-cells are predominantly recruited from the pre-existing cross-reactive memory and not the naive repertoires.

### Clonotype Diversity of Pre-existing Influenza-Specific CM T-Cells Correlates With the Serological Response to Vaccination

**Figure 4:**
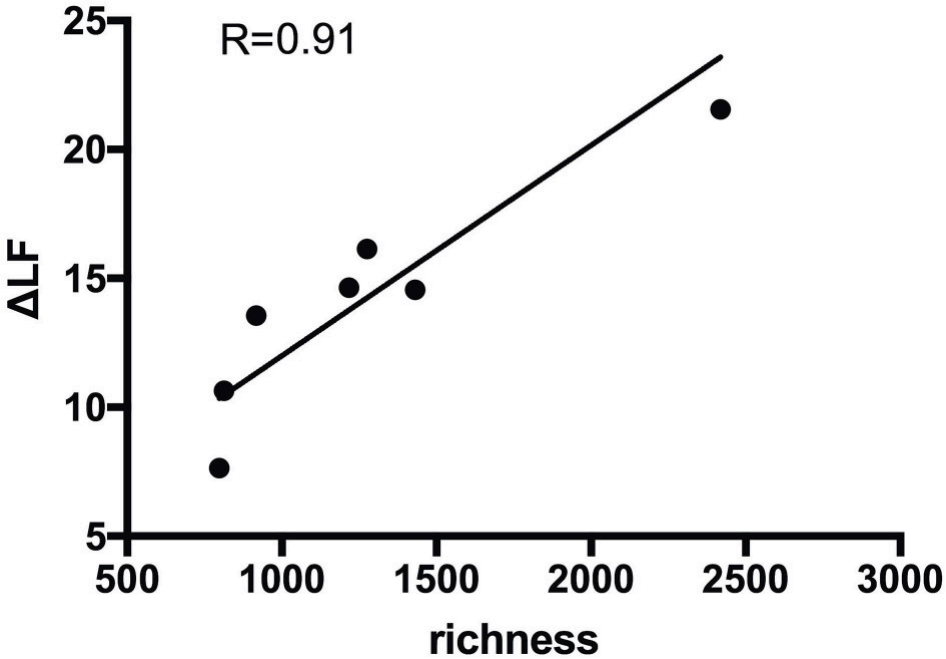
Clonotype richness of the cross-reactive vaccine-specific CM T-cells at baseline significantly correlates with the level of serological response to the previously unseen viral strains. Pearson correlation analysis between the clonotype richness of influenza-specific CM CD4 T-cell subsets (*n* = 7) at baseline and vaccination efficacy in the serologically unexperienced cohort. Clonotype richness of the influenza-specific T-cells was assessed as the number of unique clones per subset of normalized size (40,000 arbitrarily sampled raw sequencing reads according to the size of the smallest analyzed population). R, Pearson correlation coefficient. The line represents the best linear fit.

Clonotype richness/diversity is a prerequisite for an antigen-specific T-cell population to recognize broad array of pathogenic epitopes, since T-cells targeting numerous epitopes are more effective at combating the pathogens. To assess whether this feature of influenza-specific T-cells played a role in the vaccination efficacy we analyzed the correlation between the clonotype richness and the antibody titer change in the HI-negative group. As group size drastically influences diversity, the analyzed samples were first normalized to equal size. Next, sequence reads were grouped according to the clonal identity and the number of unique clones was defined as a measure of the sample richness. Our analyses revealed that the baseline richness of influenza-specific CM helper T-cells strongly correlated with serological outcome of vaccination in the HI-negative group (Figure 4; Pearson *R* = 0.91, adjusted *p* = 0.006). Clonotype diversity of further influenza-specific populations and time points revealed no correlation to the serology change. The detailed clonotype composition of CM T-helper cell at baseline in HI-negative group is presented in Table S2.

## Discussion

Influenza results in the formation of a long-term immunity that can sometimes last lifelong (4, 37). Contacts with previously seen epitopes lead to memory activation and fast pathogen clearance. However, due to antigenic drift and shift new viral strains are constantly created that can escape pre-existing antibodies and T-cells. In this case the recruitment of naive T- and B-cells is necessary for the efficient eradication of novel viruses. Not all influenza virus components mutate with equal pace with HA and NA showing the highest mutation rate (38, 39). This results in a mixed immune response to both conserved and previously unseen viral epitopes. For several pathogens, including influenza, there are concerns that the pre-existing humoral and cellular immunity can hamper the response against novel strains and skew the response against epitopes from previous encounters. This phenomenon known as original antigenic sin (OAS) was linked mostly to the pre-existing antibodies and described in numerous infections, including influenza (31, 40–44). One of the proposed mechanisms suggested the epitope masking by the pre-existing antibodies resulting in the inhibited recruitment of naive B-cells and skewed induction of memory B-cells from the previous encounters (45–47). The role of helper T-cells in the development of OAS in humans, however, was hardly addressed due to sampling and technological limitations. Original data from the Dengue virus and mouse LCMV studies pointed out on the cross-reactive T-cell memory among the reasons of the failed immunity (21, 22). Various animal studies confirmed this concept (40, 41, 48). However, new data revealed CD4+ memory specific to highly conserved internal influenza virus proteins as a protection correlate in human influenza infection (27).

Here, we analyzed the role of the pre-existing T-helper memory in the vaccination against previously unseen influenza strains. In order to exclude influence of immune senescence on the vaccination efficacy and decrease the chance of previous contacts with the vaccine strains, individuals younger than 65 were studied (49, 50). As the vaccine strains were previously circulating, we first applied anamnestic approach to exclude cases of overt infection as well as vaccination in the previous 3 years. As half of the study participants revealed vaccine-specific titers, these can be due to either subclinical/identified influenza or contacts with the virus for longer than the defined time window. Alternatively, this might reflect cross-reactive humoral immunity as broadly cross-reactive antibodies against numerous influenza strains were lately described (51, 52). Thus, final cohort definition relied on baseline serological status and serologically naive group was defined by absent vaccine-specific titers before vaccination.

The analysis of B-cell kinetics post vaccination revealed a strong increase of PB frequencies. Though the antigen specificity of the B-cell subsets was not assessed, the PB rise is most probably due to influenza-specific cells. It was previously shown, that up to 80% of PB at day 7 post vaccination are vaccine-specific (35). Furthermore, HI-positive cohort showed lower PB rise at the peak of response as compared to HI-negative one. This might be attributed to the pre-existing influenza-specific antibodies that dampen influenza-specific B-cell responses post vaccination (34). Still, even with lower PB increase the HI-positive group revealed protective antibody titers.

Using multiparameter flow cytometry and NGS-based clonotyping, we addressed the role of pre-existing helper T-cells in the early process of vaccine-specific memory formation. As MHC-class II tetramers are very limited and restrict cell analysis to a handful of epitopes and HLA-allele, we applied *ex vivo* stimulation and employed cytokine-independent analysis of antigen-specific helper T-cells (36, 53). Of notice, all subjects showed pre-existing memory T-cells at baseline, both serologically exposed and unexperienced, suggesting cross-reactive memory to conserved vaccine components and/or third-party antigens. As split vaccine was used for stimulation of influenza-specific T-cells, not only HA- and NA-specific but also T-cells with other specificities (including internal proteins NP and M1) were analyzed. The serology analysis utilizing HI-titers focused on antibodies targeting HA-antigens as these antibodies prevent hemagglutination induced by influenza hemagglutinins. However, even though present before vaccination and included into the analysis, the NP- and M1-specific T-cells (as well as other specificities against conserved antigens) are very unlikely to influence hemagglutinin-specific neutralizing titers as T- and B-cell-specific epitopes must be physically linked for efficient T-cell help (54, 55). In fact, it would be interesting to further clarify the influence of pre-existing NP- and M1-specific T-helper cell as cytotoxic T-cell with these specificities were associated with reduced influenza severity (13). Notably, memory T-helper cells specific to third-party microbial/environmental antigens were shown to be cross-reactive to influenza (as well as HIV) that were boosted after vaccination. As newborns showed only naive T-cells with these specificities it was linked to increased infection vulnerability (56).

On the clonal level we showed that in the serologically unexperienced group the vaccine-induced T-cells are recruited mostly from the pre-existing cross-reactive T-cell memory. Even though naive-derived T-cells also contributed to post vaccine-induced response, the clonotype share of naive-derived cells was neglectable as compared to pre-existing memory. To our knowledge this is the first report comparing contribution of pre-existing memory and naive T-cells in influenza vaccination. Our findings are in line with recent reports from animal LIV influenza vaccination showing that the pre-existing cross-reactive CD8 T-cell memory hampered recruitment of naive specificities (23, 57). Another study suggested that the subdominant heterotypic CD8 clonalities suppress naive precursors (58). Further mouse influenza studies revealed that cross-reactive memory specific to conserved epitopes inhibited expansion of naive specificities (25, 59). Another very recent report on whole blood clonotype analysis did not reveal significant clonotype changes after influenza vaccination suggesting that only a limited number of T-cells was recruited in the course of vaccination which was not visible on a global scale (60). Our findings show that in serologically unexperienced individuals, the pre-existing cross-reactive T-cells provide sufficient help to naive B-cells. There is still a small hypothetical possibility, that even with lacking HI-titers low levels of HA-specific non-neutralizing antibodies were present at baseline stemming from the cross-reactive memory B-cells specific to close viral strains. These cross-reactive B-cells would eventually develop highly neutralizing antibodies through somatic hypermutation. However, regardless of the source of HI-titers, either naive of cross-reactive memory B-cells, the pre-existing T-cells are helpful in generating protective antibody titers with no or limited recruitment of naive T-cells.

We detected vaccine-specific T-cells with naive phenotype not only at baseline but also in follow-up, a phenomenon not unique to influenza. Recently, we reported on high frequencies of *A. fumigatus*-specific helper T-cells with naive phenotype (31). Even though *Aspergillus* represents a ubiquitous pathogen that constantly tackles the immune system, the substantial amount of fungus-specific T-cells still remain in the naive pool. This “dispensability” of naive T-cells might be another hint on sufficient help from the pre-formed T-cell memory.

Our analysis revealed that the pre-existing influenza-specific helper T-cells with CM phenotype at baseline strongly correlate with the serological response. To our knowledge this is the first report on the differentiation status of influenza-specific CD4 T-cells and vaccination efficacy. The post vaccination expansions of influenza-specific IFNg-producing T-helper cells as measured by Elispot were shown to tightly correlate with increase of neutralizing antibodies. However, no correlation was found between serology and the pre-existing cross-reactive T-cells at baseline (54, 61). Here, influenza-specific T-cells were analyzed independently of cytokine-producing capacities in with markers allowing analysis of differentiation status.

Another, yet unresolved question is to which extent the diversity of the antigen-specific T-cells is important for an efficient immune response. Few reports addressed the role of clonotype diversity of antigen-specific T-cells in immune response and specifically influenza vaccination (62, 63). As more diverse clonotypes cover higher array of antigens, this should more efficiently target a pathogen. We showed strong correlation between the richness of pre-existing influenza-specific CM helper T-cells and the humoral response to previously unseen influenza vaccine strains. To our knowledge this is the first report on the repertoire diversity of pre-existing influenza-specific T-cells and vaccination efficacy. In line with our results are the data showing impaired influenza-specific response by restricted diversity CD8 T-cells in mouse model (64). Another indication on the role of clonotype composition comes from the human CMV setting, showing inverse correlation between the breadth of CMV-specific clonotype and antibody titers (65). Our data are strongly corroborated by the analysis of circulating follicular T-helper cells (cTfh) in influenza vaccination that revealed strong correlation between an increased cTfh-clonality and rise of peripheral plasmablast cells post vaccination (62). However, no link between cTfh-clonality at baseline and vaccination efficacy was found. As baseline cTfh encompass limited specificities to previously encountered influenza strains, further T-helper subsets with differing specificities join cTfh-pool upon vaccination and provide efficient help to influenza-specific B-cells.

Although the role of pre-existing T-cells in the generation of sufficient humoral immunity needs further analysis, several authors suggest that the pre-existing memory T-cells can be of great practical importance in vaccination (66, 67). In mouse models, immunization with inactivated influenza viruses in the presence of cholera toxin was reported to increase cross-reactivity and enhance levels of neutralizing antibodies (68). Currently, numerous studies in humans try to piggyback cellular immunity to standard vaccines (diphtheria, tetanus, and pertussis) in order to improve vaccination efficacy in risk groups (69–71).

One limitation of the current study is the lack of patients with failed vaccination. Additional studies on a cohort with low or no response to vaccine in scenarios with or without pre-existing antibodies would help to elucidate the role of pre-existing immunity, either beneficial or detrimental, in this process.

Taken together, our study demonstrates an important role of pre-existing memory T-cells in the generation of vaccine-specific humoral immunity to previously unseen strains. While naive vaccine-specific T-cells could be detected prior and after vaccine application independently of serological status, these cells were not recruited in the formation of vaccine-specific cellular memory as demonstrated by NGS and multiparameter flow cytometry. Our findings suggest that T-cell memory from previous encounters with close influenza strains provides sufficient help to naive B-cells specific to previously unseen viral strains and that the extent of previous encounters is beneficial in terms of vaccine-induced antibody titers.

## Data Availability

The datasets generated for this study can be found in Sequence Read Archive, PRJNA445234.

## Ethics Statement

The study was conducted after approval by the local ethics board (Charité University Medicine Berlin, EA1/175/11). All adult subjects provided informed written consent.

## Author Contributions

Conceptualization: MN, AT, and NB; Methodology: MN, BS, AUN, AT, and NB; Software: US, FM, KJ, SR, and LK; Formal analysis: MN, FM, US, LG, SR, and LK; Investigation: MN, US, BS, JH and SK; Resources: BS, AUN, TW, PR, AT, JH, and NB; Data curation: FM, SR, and LK; Writing—original draft: MN; Visualization: MN, FM; Supervision: MN, PR, AT, NB; Funding acquisition: AUN, AT, SR, and NB.

### Conflict of Interest Statement

The authors declare that the research was conducted in the absence of any commercial or financial relationships that could be construed as a potential conflict of interest.
