# Supplementary material for: The Role of Pre-existing Cross-Reactive Central Memory CD4 T-Cells in Vaccination With Previously Unseen Influenza Strains
Source: Front Immunol. 2019 Apr 4;10:593. doi: 10.3389/fimmu.2019.00593 (PMC6458262; doi:10.3389/fimmu.2019.00593)
Supplement: Supplementary file 1 [file Data_Sheet_1.docx]

**Supplementary Methods**

**Parameters applied for the repertoire analyses using IMSEQ 1.1.0:**

- reference (-ref): Homo.Sapiens.TRB.fa
- maximum error rate allowed within the V segment alignment (-ev): 0.3
- maximum error rate allowed within the J segment alignment (-ej): 0.25
- maximum number of errors when match the J core fragments (-jce): 3
- minimum average read phred score (-mq): 30

**Supplementary Tables and Figures**

**Table S1.** Overview of the sorted flu-specific CD4 helper T-cell samples with the outcome of the NGS-based clonotype analysis.

| **Donor** | **Day** | **Marker** | **Cells** | **Functional TCRβ** |
| --- | --- | --- | --- | --- |
| #30 | D0 | CM | 1500 | 304309 |
| #30 | D0 | Eff | 2100 | 404913 |
| #30 | D0 | naive | 174 | 208001 |
| #30 | D14 | CM | 2000 | 337942 |
| #30 | D14 | Eff | 2600 | 403647 |
| #30 | D21 | CM | 5600 | 229086 |
| #30 | D21 | Eff | 5600 | 312024 |
| #30 | D7 | CM | 2300 | 337692 |
| #30 | D7 | Eff | 2700 | 422465 |
| #38 | D0 | CM | 2170 | 771751 |
| #38 | D0 | Eff | 4200 | 1125106 |
| #38 | D0 | naive | 300 | 847690 |
| #38 | D14 | CM | 11000 | 781344 |
| #38 | D14 | Eff | 11700 | 1109292 |
| #38 | D21 | CM | 15800 | 1054079 |
| #38 | D21 | Eff | 14800 | 829860 |
| #38 | D7 | CM | 22400 | 757932 |
| #38 | D7 | Eff | 10300 | 827749 |
| #39 | D0 | CM | 3120 | 979427 |
| #39 | D0 | Eff | 150 | 616859 |
| #39 | D0 | naive | 300 | 637932 |
| #39 | D14 | CM | 11200 | 258499 |
| #39 | D14 | Eff | 460 | 1056074 |
| #39 | D7 | CM | 12900 | 1489823 |
| #39 | D7 | Eff | 1020 | 1254435 |
| #40 | D0 | CM | 1958 | 241520 |
| #40 | D0 | Eff | 1137 | 74471 |
| #40 | D14 | CM | 1900 | 156883 |
| #40 | D14 | Eff | 1200 | 150307 |
| #40 | D21 | CM | 4100 | 182635 |
| #40 | D21 | Eff | 1000 | 58945 |
| #40 | D7 | CM | 4100 | 186295 |
| #40 | D7 | Eff | 970 | 101190 |
| #42 | D0 | CM | 2900 | 813236 |
| #42 | D0 | Eff | 1200 | 1167510 |
| #42 | D0 | naive | 500 | 1091416 |
| #42 | D14 | CM | 2300 | 764339 |
| #42 | D14 | Eff | 410 | 1764564 |
| #42 | D21 | CM | 4300 | 901336 |
| #42 | D21 | Eff | 630 | 1356869 |
| #42 | D7 | CM | 12750 | 673925 |
| #42 | D7 | Eff | 1150 | 887618 |
| #51 | D0 | CM | 8800 | 174720 |
| #51 | D0 | Eff | 300 | 4386771 |
| #51 | D0 | naive | 600 | 127455 |
| #51 | D14 | CM | 11670 | 117659 |
| #51 | D14 | Eff | 3330 | 200572 |
| #51 | D21 | CM | 3697 | 1506259 |
| #51 | D21 | Eff | 503 | 253018 |
| #51 | D7 | CM | 57650 | 456015 |
| #51 | D7 | Eff | 12100 | 665600 |
| #53 | D0 | CM | 7200 | 846474 |
| #53 | D0 | Eff | 1800 | 827418 |
| #53 | D0 | naive | 1780 | 1086491 |
| #53 | D14 | CM | 14650 | 2042942 |
| #53 | D14 | Eff | 3205 | 583922 |
| #53 | D21 | CM | 3026 | 143136 |
| #53 | D21 | Eff | 645 | 303289 |
| #53 | D7 | CM | 55590 | 490173 |
| #53 | D7 | Eff | 12100 | 1630115 |

**Table S2.** Clonotype composition of influenza-specific central memory CD4 T-cells in HI-negative cohort at baseline.

| **Donor** | **V-beta** | **CDR3** | **J-beta** | **Frequency (%)** |
| --- | --- | --- | --- | --- |
| #30 | 10-3 | CAISEANSPLHF | 1-6 | 3.34 |
|  | 6-1 | CATQATRDSGYEQYF | 2-7 | 3.32 |
|  | 6-6 | CASSYSFQGDGVKNSPLHF | 1-6 | 3.03 |
|  | 6-1 | CASRLAGGGGYGYTF | 1-2 | 2.67 |
|  | 6-2/3 | CASSPPGPQSNQPQHF | 1-5 | 2.37 |
| #38 | 19 | CASRGPTRNNEQFF | 2-1 | 11.18 |
|  | 10-3 | CAISEAYTGELFF | 2-2 | 5.95 |
|  | 19 | CASSIEIAGVRDEQFF | 2-1 | 4.64 |
|  | 6-2/3 | CASSSPFRVSYNEQFF | 2-1 | 3.94 |
|  | 6-1 | CASSESGDTIYF | 1-3 | 3.63 |
| #39 | 6-1 | CASSVDLIWEQFF | 2-1 | 11.55 |
|  | 7-2 | CASSLTGTGDQPQHF | 1-5 | 5.50 |
|  | 29-1 | CSVTISIGAGELFF | 2-2 | 4.97 |
|  | 29-1 | CSVEVVRSTRPRKNQPQHF | 1-5 | 4.79 |
|  | 19 | CASSISVGQGRGHQPQHF | 1-5 | 4.48 |
| #40 | 6-1 | CASSEALTGFAEAFF | 1-1 | 12.31 |
|  | 6-1 | CASRYNTEAFF | 1-1 | 6.91 |
|  | 6-2/3 | CASSYSGIDTQYF | 2-3 | 5.07 |
|  | 19 | CASSAQGGLYNEQFF | 2-1 | 4.75 |
|  | 10-3 | CAIRGTVSGELFF | 2-2 | 4.59 |
| #42 | 6-1 | CASRRGREGWANQPQHF | 1-5 | 5.43 |
|  | 6-1 | CASRGQARGYTF | 1-2 | 4.04 |
|  | 18 | CASSPPNPSPTDTQYF | 2-3 | 3.19 |
|  | 13 | CASSLFGQYNSPLHF | 1-6 | 3.03 |
|  | 7-2 | CASSPRRADTGELFF | 2-2 | 2.10 |
| #51 | 18 | CASSSQRRQPQHF | 1-5 | 50.84 |
|  | 18 | CASSPTGGTGSRSVRAFF | 1-1 | 23.88 |
|  | 30 | CAWSRLAGPQDTQYF | 2-3 | 6.69 |
|  | 6-5 | CASRRTSGGPDTQYF | 2-3 | 2.24 |
|  | 18 | CASSSQRRQPQQF | 1-5 | 2.10 |
| #53 | 27 | CASSTPARLNEQFF | 2-1 | 1.26 |
|  | 6-1 | CASSDGGSYEQYF | 2-7 | 1.24 |
|  | 6-1 | CASSSGDSYEQYF | 2-7 | 1.16 |
|  | 6-2/3 | CASSYSGGPRLGGPLHF | 1-6 | 1.11 |
|  | 7-2 | CASSLGPLSGASYNEQFF | 2-1 | 0.77 |

Five most abundant clones with the utilized V-beta-, J-beta-genes and amino acid CDR3 sequence with the corresponding clonal abundance are presented.

**Fig S1.** Multiparameter flow cytometric analysis of peripheral blood B-cells.

Gating strategy implemented during *ex vivo* analysis of peripheral blood plasmablasts.

**Fig S2.** Multiparameter flow cytometric analysis of vaccine-specific T-helper cells.

Gating strategy implemented during *ex vivo* analysis and FACS-sorting of vaccine-specific helper T-cells.

**Fig S3**. Vaccine-specific naive T-cells produce no cytokines or low level of IL2 in contrast to pre-existing cross-reactive memory T-cells producing high levels of effector cytokines.

Different cytokine-secreting capacities of influenza-specific CD4 T-cells with different developmental status at baseline are shown. Each point represents one analyzed population. Frequencies of cytokine-producing cells within parental influenza-specific T-cell subsets are shown. (A) represents cells producing either low amounts of IL2 only or no analyzed cytokines. (B) represents cells producing at least one analyzed effector cytokine alone or in combination (TNFa, IFNg, IL4 or IL17).
